# Opiate users' knowledge about overdose prevention and naloxone in New York City: a focus group study

**DOI:** 10.1186/1477-7517-3-19

**Published:** 2006-07-05

**Authors:** Nancy Worthington, Tinka Markham Piper, Sandro Galea, David Rosenthal

**Affiliations:** 1 Lower East Side Harm Reduction Center, New York, NY 10002, USA; 2 Center for Urban Epidemiologic Studies, New York Academy of Medicine, New York, NY 10029, USA; 3 Department of Epidemiology, University of Michigan School of Public Health, Ann Arbor, MI, 48104, USA

## Abstract

**Background:**

Drug-induced and drug-related deaths have been increasing for the past decade throughout the US. In NYC, drug overdose accounts for nearly 900 deaths per year, a figure that exceeds the number of deaths each year from homicide. Naloxone, a highly effective opiate antagonist, has for decades been used by doctors and paramedics during emergency resuscitation after an opiate overdose. Following the lead of programs in Europe and the US who have successfully distributed take-home naloxone, the Overdose Prevention and Reversal Program at the Lower East Side Harm Reduction Center (LESHRC) has started providing a similar resource for opiate users in NYC. Participants in the program receive a prescription for two doses of naloxone, with refills as needed, and comprehensive training to reduce overdose risk, administer naloxone, perform rescue breathing, and call 911. As of September 2005, 204 participants have received naloxone and been trained, and 40 have revived an overdosing friend or family member. While naloxone accessibility stands as a proven life-saving measure, some opiates users at LESHRC have expressed only minimal interest in naloxone use, due to past experiences and common misconceptions.

**Methods:**

In order to improve the naloxone distribution program two focus groups were conducted in December 2004 with 13 opiate users at LESHRC to examine knowledge about overdose and overdose prevention. The focus groups assessed participants' (i) experiences with overdose response, specifically naloxone (ii) understanding and perceptions of naloxone, (iii) comfort level with naloxone administration and (iv) feedback about increasing the visibility and desirability of the naloxone distribution program.

**Results:**

Analyses suggest that there is both support for and resistance to take-home naloxone, marked by enthusiasm for its potential role in reviving an overdosing individual, numerous misconceptions and negative views of its impact and use.

**Conclusion:**

Focus group results will be used to increase participation in the program and reshape perceptions about naloxone among opiate users, also targeting those already prescribed naloxone to increase their comfort using it. Since NYC is advancing toward a citywide naloxone distribution program, the LESHRC program will play an important role in establishing protocol for effective and wide-reaching naloxone availability.

## Background

Drug-induced mortality and morbidity have been increasing throughout the United States over the past decade. According to the Drug Abuse Warning Network, overdose-related deaths in 30 US metropolitan areas rose from 5,000 in 1996 to 6,400 in 2002 [1]. As a result, for many opiate users, experiencing overdose is a regular part of life. Studies in Australia and the UK indicate that half to two-thirds of sampled drug users had themselves previously survived overdose, and a considerably greater number had witnessed overdose by another drug user [2,3]. Overall, regularly injecting heroin users face annual mortality rates of two percent, a rate that is half attributable to overdose, and six to 20 times the mortality rate expected in non-drug-using peers [4-10].

In New York City, an estimated 900 opiate users die from overdose each year, a figure that exceeds the number of deaths from homicide [11]. Also in NYC, drug consumption currently ranks among the five leading causes of mortality in 15–54 year-olds, and comprises up to nine percent of hospitalization in some neighborhoods [12-14].

Opiates produce their effect by binding with neural receptor sites at the expense of proper breathing function. In the event of increased opiate consumption, breathing is diminished and loss of consciousness occurs. Due to unpredictable variables in drug strength or supply, or reduced tolerance level after periods of cessation of use, opiate overdose may be unavoidable, even if individuals take preventative measures (i.e. knowing tolerance and drug source, injecting slowly, not using alone). By no means, however, must death be a necessary outcome. Overdose is rarely instantaneous and usually happens in the presence of others [10,15-18]. Bystanders may not seek professional medical assistance due to fear of police arrest, but can provide basic, effective care: mouth-to-mouth resuscitation and naloxone hydrochloride, an opiate antagonist also known as narcan. Administered most commonly through injection, naloxone displaces opiates at the receptor site, thereby restoring breathing and consciousness within minutes [19]. Physicians and emergency medical personnel in the US and throughout the world have for decades been using naloxone to treat overdose patients. Few observed complications have been reported, and existing reports of complications may be unfounded [20]. While the administration of increased doses of naloxone may incite withdrawal symptoms in some patients who are opiate dependent, the drug is otherwise harmless, exhibiting no potential for abuse and no psychopharmacological effects [21].

Naloxone has been sold over the counter in Italian pharmacies for more than ten years, and distributed to peer networks of opiate users by European and U.S. harm reduction programs since 1995. The Chicago Recovery Alliance (CRA), a national leader in the field, has operated one of the largest naloxone distribution programs to date. In 2002, CRA equipped over 1,600 people with naloxone, resulting in 115 lives saved and a 20 percent reduction in fatal overdose, the first time in years this figure was in decline [22]. San Francisco, Baltimore, and parts of New Mexico have also distributed naloxone to opiate users through needle exchange and methadone maintenance programs.

Similar work has recently begun at the Lower East Side Harm Reduction Center (LESHRC) in NYC through an on-site intervention called the Overdose Prevention and Reversal Program. LESHRC serves over 9,000 opiate users annually with clean needles and other support services, and is the first in NYC to distribute naloxone to its program participants. Recruiting opiate users as individuals, pairs, or groups, trained LESHRC staff and volunteers provide instruction in overdose risk and prevention, naloxone administration, calling 911, follow-up care, and interfacing with police. The program aims to 1) decrease the alarmingly high incidence of overdose-related death; 2) increase overdose awareness and preparedness; 3) encourage individuals to reflect upon personal overdose risk; and 4) facilitate dialogue between drug using partners regarding proper overdose response. As of September 2005, naloxone has been prescribed to over 204 LESHRC program participants, and 40 cases of successful overdose reversal with the use of peer-administered naloxone have been reported.

Current literature on opiate overdose has recognized the importance of naloxone distribution as an effective harm reduction tool. In the fall of 2004, a group of researchers and service providers from the Center for Urban Epidemiologic Studies (CUES) at the New York Academy of Medicine and LESHRC looked to build upon this recommendation. They conducted focus groups with opiate users from LESHRC to assess knowledge of overdose, overdose prevention, and overdose response, as well as attitudes about naloxone, naloxone experiences and support for its availability as a take-home medication. Because only few studies have asked opiate users to address topics specific to naloxone, the goal is to contribute to literature on opiate overdose by offering first-hand accounts of naloxone experience. Focus group results will be used to increase participation in the program, reshape perceptions about naloxone among opiate users, and encourage naloxone comfort among those who carry it as a result of the program.

## Methods

In December of 2004, two focus groups were convened at LESHRC. Study participants were recruited by means of in-house flyers, word of mouth, and referral by LESHRC staff members. Eligibility for the first focus group was determined by opiate use; for the second, completion of the Overdose Prevention and Reversal Program and receipt of naloxone. All study participants were over the age of 18. The first focus group had eight participants, three of whom were African-American females; the remaining five included one African-American male, one Hispanic male, and three White males. In the second focus group, there was a total of five participants, one African-American male and four White males. Two trained CUES and LESHRC staff members facilitated the first group using an open-ended question guide, which accessed participants' 1) experiences with overdose response, specifically naloxone; 2) understanding and perceptions of naloxone; 3) potential comfort level with naloxone administration; and 4) feedback about increasing the visibility and desirability of a naloxone distribution program. The second focus group was conducted by the above LESHRC staff member and a LESHRC program director using the same question guide, along with additional questions that addressed prior experience using naloxone on an overdosing friend, to examine naloxone comfort.

The focus groups were audio taped and later transcribed. To ensure anonymity, names were not recorded and tapes were destroyed immediately after transcription. Study participants received $15 compensation as well as transportation reimbursement. Group participation was voluntary, and study participants were welcome to decline from further participation at any point in time. Study goals and study protocol were clearly outlined at the start of each group, followed by the collection of verbal consent from each study participant. The themes presented in this paper emerged from the focus groups transcripts and were derived by two of the authors using a coding system which included open and axial coding. During open coding, each coder independently 1) studied the textual material; 2) pinpointed quotes recurring throughout the text; 3) examined them vis-à-vis related or contradictory quotes also in the text; and 4) organized them into major themes. As part of axial coding, the coders compared notes, negotiating which themes provided the richest, most saturated understanding of the study participants' knowledge about overdose and naloxone.

## Results

Participants in both focus groups expressed mixed feelings about naloxone in the context of overdose, including some hesitation to its distribution for take-home use. In our review of transcripts, we identified four major themes to capture the overall views of participants: 1) support for naloxone as a lifesaving measure; 2) challenges of administering naloxone during an overdose; 3) fear of dopesickness; and 4) fear of police arrest at the scene of an overdose after naloxone administration.

### Naloxone as lifesaving measure

Study participants unanimously recognized the potential role of naloxone in successfully reviving someone from an unconscious, overdose-induced state. Not surprisingly, most enthusiastic were participants who had already completed the Overdose Prevention and Reversal Program and received naloxone. As one participant described:

"This particular program with the naloxone...gives me a feeling of, of security. And not so much for myself, because...to tell you, to be honest, I've been using...heroin now for close to 30 something years and I have never once overdosed... However, I have a lot of friends, and a lot of my close friends...are also users, so...it gives me a feeling of security for them. To be able to help them, just in case one of them goes off the deep end...and overdoses... At least...I feel like a guardian angel, I guess."

These words demonstrated both an understanding of the breadth of fatal overdose – that is, any drug user is at risk – as well as a personal commitment to saving the life of a friend, however possible. Another participant expressed similar sentiment. She shared her experiences intervening in overdose scenarios over the years. Compared to the unproven and potentially dangerous resuscitation methods such as causing pain and applying ice, she described naloxone as "such a godsend" because she now "can give 'em that before the ambulance" arrives. The administration of naloxone to an overdose victim while awaiting more comprehensive medical care was imperative to this participant, considering both the urgency of a non-breathing individual, and the fact that ambulances are sometimes not as quickly dispatched to overdose calls. She explained, "A lot of times, fifteen, 20 minutes, if the ambulance doesn't show up, [the overdosed person] could be dead." Naloxone provided her not a substitute for calling 911 but a sense of security while awaiting an ambulance.

Non-naloxone-holding participants also voiced support for the potential role of naloxone to revive someone. However, they pointed to its merit as a necessary step only when other attempts proved unsuccessful and the consequence of death would be too much to bear. One participant stated:

"Narcan is good, when you're like, it's the last resort. I mean, you can't get them up, you put 'em in the shower, you rubbed ice on their scrotum, you've given them mouth-to-mouth resuscitation, you've pumped their chest, you've tried."

Another participant confirmed that he would "rather have narcan than die," despite the unpleasant symptoms of withdrawal that sometimes accompany its use. These participants, in particular, were clear about their support for naloxone, and furthermore, qualified the exact circumstances in which they would use it.

### Challenges of administering naloxone

Accounts of naloxone perception and experience were not entirely favorable. Participants reported challenges and fears when they reflected upon personal experiences of naloxone being administered to them or their administering it to others.

An overdose situation can be scary, chaotic, and emotionally traumatic. Bystanders untrained in proper overdose response may become paralyzed with fear or attempt to revive the person using less effective measures. In anticipation of police involvement, others may be overwhelmed with securing their own safety, frantically discarding evidence of drugs or drug paraphernalia, or planning their escape in fear of being charged with manslaughter. The sight of an overdosing friend or family member can also be distressful. The stress of an overdose situation was clearly described by focus group participants with extensive overdose experience. As one participant put it:

"Every time I've been in the situation where someone ODs, it's a panic, and ...I've always kept my cool, but everybody else around and yellin' and screamin'...and losin' their head, and...runnin' round like a chicken with their head cut off...they're scared for this person's life."

When naloxone is available, the situation is not necessarily improved. Among focus group participants, only one reported administering naloxone to an overdosing friend, who was then revived. He described the situation as hectic, himself struggling to remain calm enough to perform the injection with precision and ease.

"And you don't want to make a mistake, you know? You don't have to look for a vein...but...it's a very shaky...scary situation... I'm not looking for directions... You're nervous as hell!"

Administering naloxone can be even more complicated when the person trained to administer it is himself intoxicated. The same participant explained, "especially if you're messed up... and all five people are... high as a kite, you know... it's gonna be total panic." For this participant, the difficulty of administering naloxone, compounded by the fear that his intoxication level may pose additional barriers, were so profound that he was reluctant to receive a naloxone refill. Although he recognized the vital role of naloxone in the outcome of the event, he was unsure he could use it again.

### Fear of dopesickness

Dopesickness – or opiate withdrawal characterized by shaking, headache, nausea, and vomiting – was a prominent theme among study participants. Naloxone, particularly in larger doses, can incite withdrawal symptoms in opiate users. Focus group participants who had been given naloxone by emergency medical personnel described the effect as "the worst feeling in the world." In recounting his overdose experience, one participant reported being revived with only mild discomfort after a single naloxone injection, but when EMS administered a second dose, the physical result was unbearable:

"I was COLD... I was SWEATIN'...I was freezin'...like somebody just took the plug out and 'Oh, no. That pleasure is gone' ...having fever and chills at the same time... Everything hurts. Your whole body hurts. Uh, 'cause you're convulsing."

Other participants who had been given naloxone during an overdose confirmed reports of excruciating pain, citing that it was not an experience they wished to repeat.

Enduring dopesickness post-naloxone use presented further concerns for some study participants, who affirmed that if naloxone were ever used on them, they would have no choice but to use more opiates to ease the discomfort. As one participant noted, after naloxone, "Now you're ill again, so you gotta get MORE money to get high, 'cause now you're sick!" Another added, "You gonna have to go cop again... So even if you don't wanna, you're gonna go get it anyway." The perceived need to counteract withdrawal by using again highlights a common misconception. In truth, because naloxone only lasts 30 to 90 minutes after administration, any additional opiate consumption increases the chances of a subsequent overdose once the naloxone wears off. The above study participants, who had some naloxone experience but no formal training, were therefore familiar with its function and physiological effect but lacked important information that would lead to effective follow-up care.

Even some of the participants with formal naloxone training were misguided on how to proceed once administering naloxone to an overdosing friend or family member. They understood the risks of subsequent overdose with increased opiate use, but were not convinced that waiting until the naloxone wears off qualified as best practice. Speaking hypothetically about reviving someone with naloxone, one participant explained:

"If I had the money, I would think I would like to get 'em straight, but I'd be afraid he'd go right back into overdose, so I wouldn't do it. But if anything, I would give 'em a little methadone."

Although the study participant demonstrated an awareness that both dopesickness and subsequent overdose are associated risks of naloxone, his prioritizing immediate withdrawal relief could be potentially dangerous. Dopesickeness, unlike overdose, is non-fatal and in cases where naloxone has been administered, will subside without further medicating. A subsequent overdose could also be effectively reversed; however, it would require additional doses of naloxone that may be unavailable to the overdose responder.

### Fear of police

The final theme presented by the focus groups was fear of police involvement at the scene of an overdose after naloxone administration. These fears were less about individuals having drugs on them when police arrived, and more about liability if they used their naloxone on a third party, which at the time of writing was legally suspect. Such fears were compounded by past experience, in which police officers rarely elicited information before performing arrests, and furthermore, treated all drug users at the scene of an overdose as responsible parties. In reflecting upon situations where she would use naloxone, one participant stated:

"There is the police factor... So you might be more scared about the damn cops than saving someone's life. So that's the choice you gotta make. You might be facin' some serious time."

Participants who shared this view requested additional training in how to effectively communicate with police officers who arrive at the scene of an overdose, particularly if they have used their naloxone on someone without a naloxone prescription.

The desire to save another person whatever the consequence, however, overpowered these fears in the case of other participants. One participant explained, "If I see a person's life on the line, my first thought would be, the first thing, to just bring them revival." Using naloxone beyond its recommended purpose, from his view, was a risk worth taking.

## Discussion

Focus group results show that naloxone is undeniably advantageous for individuals to effectively revive an overdosing friend or family member, instead of resorting to potentially harmful and less effective methods of resuscitation. Participants' narratives also point to other considerations – the challenges of administering naloxone, fear of dopesickness, and fear of police. All of these areas reveal some benefits and challenges to naloxone training as a critical arena for overdose prevention, and offer important insights for improving overdose prevention and reversal efforts.

Support for naloxone as a lifesaving measure is shared by opiate users around the world. One study shows that 70 percent of sampled opiate users voiced support for naloxone as a take-home medication, and 49 percent reported a willingness to keep supplies on hand [23]. These results have caused some concern that witnesses to an overdose would therefore use naloxone as a substitute for calling 911 [23-25]. As indicated by focus group participants, however, naloxone is considered an acceptable last resort while awaiting the arrival of trained emergency medical personnel. Although fear of police arrest was reported as a complicating factor, no participant expressed adamant refusal to involving the medical system, suggesting that widespread availability of naloxone may not negate messages to overdose responders that follow-up medical care be standard practice for any overdose.

The commitment to being a most effective overdose responder, however, has its drawbacks. As indicated by some study participants, the primary motivation for enrolling in a naloxone distribution program was to help an overdosing friend or family member, which may mean that personal perceptions of overdose risk are being largely ignored or overlooked. Another study found that approximately half of the study sample reported an overdose over the course of six months, yet almost three-quarter of the respondents had rarely or never worried about the possibility of overdosing during the same time period [24]. Participants in our study, and opiate users in general, may need more encouragement to reflect upon personal overdose risk, which ultimately may be the most effective measure to save lives.

The challenges of take-home naloxone (i.e. the impact of panic and intoxication on successful naloxone administration) reported by some of the study participants and in other studies [26,27] offer another important insight. Considering that individuals could gain comfort and confidence using naloxone through practice or follow-up training, naloxone distribution programs may consider arranging multiple visits with enrolled participants to review protocol, practice role plays of naloxone administration, provide support, and address fears.

Also noteworthy was the aversion some study participants had to past or anticipated naloxone experiences. The study participant who had used naloxone on a friend described the events as challenging, stressful, and emotionally upsetting, and the others who had received naloxone, or even only heard of it, were discouraged by the potential for dopesickness post-administration. This refutes concerns that take-home naloxone could encourage riskier drug-taking activity in opiate users who would be therefore comfortable using beyond their tolerance, knowing a friend could quickly revive them in the event they overdosed [28,29].

The threat of dopesickness, however, could effect participant interest in naloxone distribution programs. In particular, fears of dopesickness may have been one of the factors that deterred participants from immediately enrolling in the Overdose Prevention and Reversal Program where naloxone distribution was publicized as the primary aim. Although these study participants reported that they would rather receive naloxone than die, they may have also been trying to avoid situations with naloxone entirely. This identifies a need to promote prevention and intervention strategies focused on overdose where naloxone is not the main feature, in order to capture the attention of opiate users who would otherwise remain disconnected.

Opiate users' strong feelings about withdrawal symptoms post-naloxone also indicates that they may ignore warnings against continued opiate consumption after an overdose reversal. Although premature re-consumption of heroin or other opiates rarely leads to a subsequent overdose once naloxone wears off [30], the issue should not be ignored. There may be a need for increased education and resources for individuals to support a friend regaining stability after an overdose event. While encouraging participants to seek medical help is one option, another may be distributing naloxone alongside controlled amounts of buphrenorphine or methadone, so they may experience some degree of immediate withdrawal relief.

Fear of police in our study revealed that participant concerns were less about potentially facing charges for possession of drugs, possession of drug paraphernalia, or manslaughter, and more about the consequences if they used their naloxone on a non-prescription holding individual. While literature confirms that drug possession and manslaughter are not major deterrents to calling 911 [3], only minimal attention has been given to fears about administering naloxone to a third party. New Mexico has most effectively addressed this issue by providing legal protection to anyone, physician or bystander, administering naloxone to opiate victims with or without naloxone prescription. To increase the comfort and feasibility of naloxone use throughout the US, state legislative bodies should take similar action steps (see appendix 1).

## Conclusion

In summary, these focus groups highlighted the strengths and weaknesses of naloxone distribution programs, as well as indicated areas for further exploration. The lessons learned are useful for several reasons. First, they merit the attention of researchers and service providers committed to improving overdose intervention strategies nationwide. Second, they provide a framework for new naloxone programming taking place in NYC. Soon after LESHRC began its naloxone program, the NYC Injection Drug User Health Alliance obtained funding for similar overdose prevention and reversal efforts, including naloxone distribution, at consenting needle exchange programs. Our study results, considering their geographic relevance, serve to inform NYC-based needle exchange programs as they continue to develop effective services for opiate users to reduce overdose risk, as well as to assist friends and family. The findings also benefit any drug- or non-drug-using individual who has connections with opiate users and may be able to intervene in a time of need. Future research will need to assess the continued viability of take-home naloxone, once naloxone programs have refined training strategies to address fear of dopesickness and police, and the challenges often associated with an overdose event.

## Competing interests

The author(s) declare that there are no competing interests.

## Authors' contributions

NW facilitated both focus groups, performed analysis, and drafted the manuscript. TMP facilitated the first focus group, performed analysis, created the manuscript outline, and helped to draft the manuscript. SG conceived of the study and helped to draft the manuscript. DR facilitated the second focus group and helped to draft the manuscript. All authors participated in coordination of the study and read and approved the final manuscript.

## Appendix 1

On August 2, 2005, Governor George Pataki signed a bill regarding opiod overdose prevention that authorizes 1) the state health commissioner to establish standards for overdose prevention programs and 2) the use of naloxone by non-medical staff in the case of an overdose. The law takes effect in April 2006. Such legislation will hopefully increase awareness of overdose prevalence and increased naloxone use in overdose prevention among opiate users.
